# Solar X-ray and EUV imager on board the FY-3E satellite

**DOI:** 10.1038/s41377-022-01023-z

**Published:** 2022-11-22

**Authors:** Bo Chen, Xiao-Xin Zhang, Ling-Ping He, Ke-Fei Song, Shi-Jie Liu, Guang-Xing Ding, Jin-Ping Dun, Jia-Wei Li, Zhao-Hui Li, Quan-Feng Guo, Hai-Feng Wang, Xiao-Dong Wang, Yun-Qi Wang, Hong-Ji Zhang, Guang Zhang, Zhen-Wei Han, Shuang Dai, Pei-Jie Zhang, Liang Sun, Yang Liu, Peng Wang, Kun Wu, Chen Tao, Shi-Lei Mao, Gui Mei, Liang Yang, Li-Heng Chen, Chun-Yang Han, Bin Huang, Yang Liu, Shuai Ren, Peng Zhou, Ze-Xi Wei, Xiao-Xue Zhang, Yue Zhang, Xin Zheng, Yang Wang, Ya Chen, Jing-Jiang Xie, Fei He, Qiao Song, Wei-Guo Zong, Xiu-Qing Hu, Peng Zhang, Jing-Song Wang, Zhong-Dong Yang

**Affiliations:** 1 Changchun Institute of Optics, Fine Mechanics and Physics, Chinese Academy of Sciences, Changchun, China; 2 State Key Laboratory of Applied Optics, Chinese Academy of Sciences, Changchun, China; 3 National Satellite Meteorological Center, China Meteorological Administration, Beijing, China; 4 Key Laboratory of Space Weather, National Center for Space Weather, China Meteorological Administration, Beijing, China; 5 Innovation Center for Fengyun Meteorological Satellite (FYSIC), Beijing, China; 6 Key Laboratory of Earth and Planetary Physics, Institute of Geology and Geophysics, Chinese Academy of Sciences, Beijing, China

**Keywords:** Imaging and sensing, X-rays

## Abstract

The solar X-ray and Extreme Ultraviolet Imager (X-EUVI), developed by the Changchun Institute of Optics, Fine Mechanics and Physics, Chinese Academy of Sciences (CIOMP), is the first space-based solar X-ray and Extreme ultraviolet (EUV) imager of China loaded on the Fengyun-3E (FY-3E) satellite supported by the China Meteorological Administration (CMA) for solar observation. Since started work on July 11, 2021, X-EUVI has obtained many solar images. The instrument employs an innovative dual-band design to monitor a much larger temperature range on the Sun, which covers 0.6–8.0 nm in the X-ray region with six channels and 19.5 nm in the EUV region. X-EUVI has a field of view of 42′, an angular resolution of 2.5″ per pixel in the EUV band and an angular resolution of 4.1″ per pixel in the X-ray band. The instrument also includes an X-ray and EUV irradiance sensor (X-EUVS) with the same bands as its imaging optics, which measures the solar irradiance and regularly calibrates the solar images. The radiometric calibration of X-EUVS on the ground has been completed, with a calibration accuracy of 12%. X-EUVI is loaded on the FY-3E satellite and rotates relative to the Sun at a uniform rate. Flat-field calibration is conducted by utilizing successive rotation solar images. The agreement between preliminarily processed X-EUVI images and SDO/AIA and Hinode/XRT images indicates that X-EUVI and the data processing algorithm operate properly and that the data from X-EUVI can be applied to the space weather forecast system of CMA and scientific investigations on solar activity.

## Introduction

The solar X-ray and Extreme Ultraviolet Imager (X-EUVI)^1,2^ is designed to cover 0.6–8.0 nm in the X-ray region and 19.5 nm in the EUV region to monitor a much larger temperature range on the Sun. The 19.5 nm line is emitted by Fe XII at approximately 1.5 × 10^6^ K, and the flare loops usually contain a large amount of material at this temperature^1^. The 0.6–8.0 nm region is emitted by different ionizations of Si, Mg, Fe and various other elements at temperatures greater than approximately 10^7^ K^3–6^. Through these working bands, X-EUVI can observe a much larger temperature range in the atmosphere of the Sun composed of the chromosphere, transition region, and corona to improve the forecast of space weather and early warnings of possible impacts on the Earth’s environment. Space-borne optical remote instruments for the Sun have been developed for more than 40 years, and many X-ray and EUV imaging instruments have been launched to study solar atmospheric dynamics. The representative instruments are described as follows.

In 1991, the Soft X-ray Telescope (SXT) of the Yohkoh mission, proposed by Japan, the USA and the UK, was launched to produce X-ray movies of flares with excellent angular and time resolutions as well as full-disk X-ray images for the general studies. The observed wavelengths were 0.3–6.0 nm, and the angular and time resolutions were 2.5” and 0.5 s, respectively^7–9^. In 2006, the X-ray Telescope (XRT) onboard the Hinode, developed by ISAS/JAXA, was launched. As the successor to the Yohkoh mission, it aims to determine the mechanisms responsible for eruptive phenomena, such as flares and coronal mass ejections (CMEs). The observed wavelengths were 0.2–20.0 nm with 9 channels, and the angular and time resolutions were 1.0” and 10 s, respectively^10,11^. The Solar X-ray Imagers (SXI), flown on the Geostationary Operational Environmental Satellites (GOES-12, GOES-13, GOES-14, GOES-15) of the National Oceanic and Atmospheric Administration (NOAA), imaged the full-disk Sun at wavelengths between 0.6 and 6.0 nm, with an angular resolution of 5” and a time resolution of 1 min^12^.

For solar observations in the EUV band, the Extreme-ultraviolet Imaging Telescope (EIT) onboard the Solar and Heliospheric Observatory (SOHO) was launched in 1995 to provide images of the corona and transition region on the solar disk^13–15^. It selected spectral emission lines of 17.1 nm (Fe IX), 19.5 nm (Fe XII), 28.4 nm (Fe XV) and 30.4 nm (He II) to provide sensitive temperature diagnostics in the range from 6 × 10^4^ to 3 × 10^6^ K. The telescope had an angular resolution of 2.6″ per pixel and a time resolution of 90 s. In 1998, the Transition Region and Coronal Explorer (TRACE) mission with three EUV imaging channels were launched to image the solar corona at 17.1, 19.5 and 28.4 nm for diagnosis of coronal plasmas between 10^5^ and 10^6^ K^16,17^. As the successor to TRACE, the National Aeronautics and Space Administration (NASA) launched the Solar Dynamics Observatory (SDO) mission in 2010, and the Atmospheric Imaging Assembly (AIA) onboard SDO has provided near-continuous monitoring of the Sun in 7 narrowband EUV channels^18,19^. The Extreme Ultraviolet Imager (EUI), equipped in the Solar Orbiter mission, was launched last year, which aims to provide full-disk EUV and high-resolution EUV and Lyman-α imaging of the solar atmosphere by imaging the three spectral lines of 17.4, 30.4 and 121.6 nm^20,21^. A summary of the parameters of these typical imagers is presented in Table 1.

**Table 1:** Summary of typical solar imagers

Imager/Year	Wavelength (nm)	Angular resolution (“)	Time resolution (s)	Spacecraft altitude (km)	Reference
Yohkoh /SXT, 1991	0.3–6.0	2.5	0.5	500	^7–9^
SOHO/EIT, 1995	17.1, 19.5, 28.4, 30.4	2.6	90	1.5 × 10^6^	^13–15^
TRACE, 1998	17–29 (3 channels)	0.5	30	627	^16,17^
Hinode/XRT, 2006	0.2–20 (9 channels)	1.0	10	650	^10,11^
GOES^a^/SXI	0.6–6.0	5.0	60	35,786	^12^
GOES^b^/SUVI	9.4–30.4 (6 channels)	2.5	0.4	35,756	^31,32^
SDO/AIA, 2010	9–34 (7 channels), UV, Vis	0.6	10	35,756	^18,19^
Solar Obiter/EUI,2021	17.4, 30.4, 121.6	4.5 (FSI), 0.5 (HRI)	10–600	0.3 AU	^20,21^
PROBA2/SWAP,2009	17.4	3.17	60	720	^33^
X-EUVI/FY-3E, 2021	0.6–8.0 (6 channels), 19.5	4.1 (X-ray), 2.5 (EUV)	7–20	836	^This paper^

^a^GOES-12, GOES-13, GOES-14, GOES-15 satellites

^b^GOES-16 and GOES-17 satellites

Benefiting from the observations by the above solar X-ray and EUV imagers, many solar atmospheric activities were discovered in the past decades^22–26^, which led to many discoveries of physical and chemical processes that occur in the Sun. The solar activity processes determine the plasma and radiation state near the Earth space and influence the state of the upper atmosphere, ionosphere and magnetic field, including the radiation belt, of the Earth. The flare is one of the most important active phenomena on the Sun. X-ray imaging observation data are particularly important for the study of high-temperature plasma in the corona (~10^7^ K), which represents the region with the strongest energy release and the core of solar flares. EUV imager can monitor low-coronal phenomena such as filament eruptions in real-time and observe the CME phenomena.

All the above instruments worked either in the EUV band or in the X-ray band^5–19^. However, dual-band observations have never been accomplished on one instrument. Since 2009, the research team of the Changchun Institute of Optics, Fine Mechanics and Physics, Chinese Academy of Sciences (CIOMP) has made a series of key technology breakthroughs and developed a solar X-ray and EUV dual-band imager prototype supported by the National Natural Science Foundation of China (NSFC). Based on the prototype, the first dual-band solar imager has been developed by CIOMP, which utilizes an innovative design covering X-ray and EUV dual bands. The equipped X-EUVS can calibrate each imaging channel of X-EUVI in orbit to obtain solar images with absolute brightness. In this paper, the design and development will be presented.

## Results

**Fig. 1:**
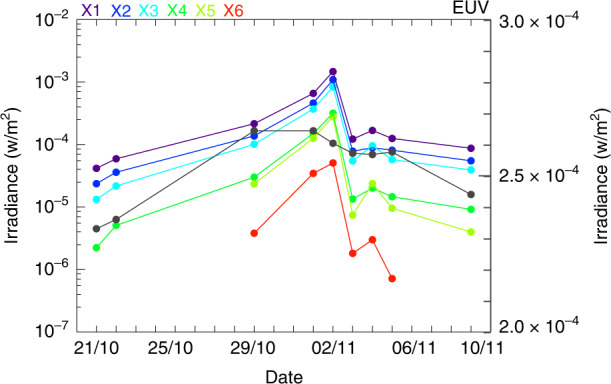
Solar irradiance variations measured by X-EUVS. EUV (black): 19.5 nm, X1: 0.6–8.0 nm, X2: 0.6–6.0 nm, X3: 0.6–5.0 nm, X4: 0.6–2.0 nm, X5: 0.6–1.6 nm, and X6: 0.6–1.2 nm

X-EUVI has been working in orbit since July 11, 2021, acquiring some solar irradiance data and many solar images. In particular, the dramatic changes in solar irradiance data and images were obtained with X-EUVI during the eruption of an M-class solar flare between October 21 and November 10 in Fig. 1. The solar irradiance increased from 8.06 × 10^−5^ to 4.04 × 10^−3^ W cm^−2^ in the X-ray bands, while in different X-ray bands, the magnifications varied. In the shortest X-ray band of 0.6–1.2 nm, the solar disk irradiance increased from 3.80 × 10^−6^ to 5.07 × 10^−5^ W cm^−2^ from October 29 to November 2, also in the 19.5 nm EUV band, the solar disk irradiance increased about 1.4 times, as shown in Fig. 1. X-EUVS was calibrated in CIOMP before launching, and the radiometric calibration accuracy was ~12%.

**Fig. 2:**
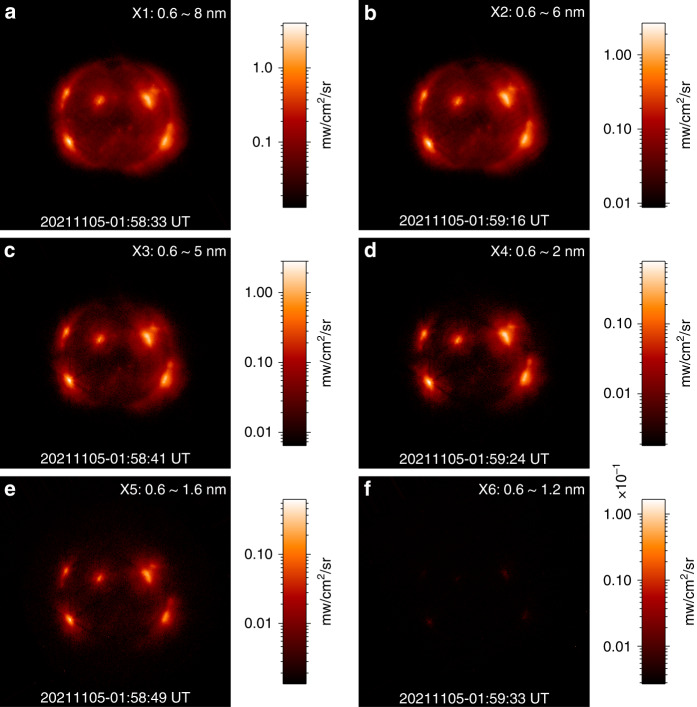
X-ray images with absolute brightness from X-EUVI. The observation time is at the bottom, and the imaging channels are at the upper right corners

**Fig. 3:**
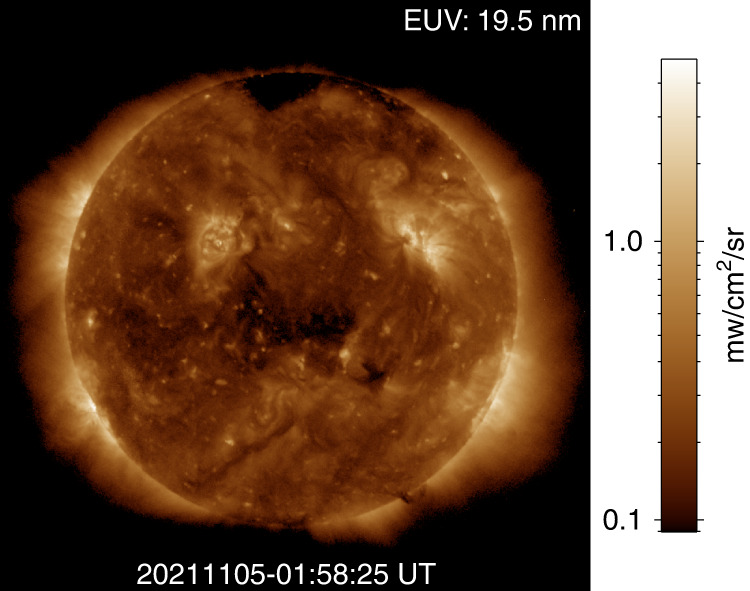
EUV 19.5 nm image with absolute brightness from X-EUVI. The notes are consistent with Fig. 2

Solar X-ray and EUV images were also acquired during solar eruptions. The brightness of X-ray images was significantly enhanced. When eruptions occur, strong X-ray flares can be observed in the images of all X-ray channels, while during solar quiet periods, only some active regions can be observed in the images of X1, X2 and X3. During this solar eruption, the intensity of the X-ray images was dramatically altered. We applied the irradiance data of X-EUVS to calibrate these images. The obtained X-ray and EUV absolute brightness images are displayed in Figs. 2 and 3.

**Fig. 4:**
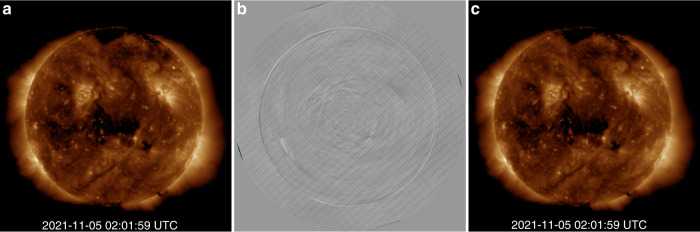
Original solar image, flat-field matrix, and corrected image. The solar image was captured by X-EUVI at 02:01 on November 5, 2021

Because the FY-3E satellite runs on a dawn-dusk sun-synchronous orbit, X-EUVI rotates around the Earth with the satellite. Based on the rotation characteristics of solar images, an improved algorithm based on KLL is applied to the on-orbit flat-field calibration of X-EUVI^24^^,26^. The KLL algorithm, proposed by Kuhn et al. in 1991, calibrates the spatial nonuniformity of an image array based on a series of offset images, and the algorithm is robust and efficiently uses information from multiple data frames to determine pixel gain variations. The flat-field matrix, the original solar image and the corrected image using the flat-field matrix are shown in Fig. 4. Because X-EUVI rotates relative to the Sun in real time, multiple rotation images can be used for flat-field calibration at any time so that accurate solar X-ray and EUV images can be obtained.

**Fig. 5:**
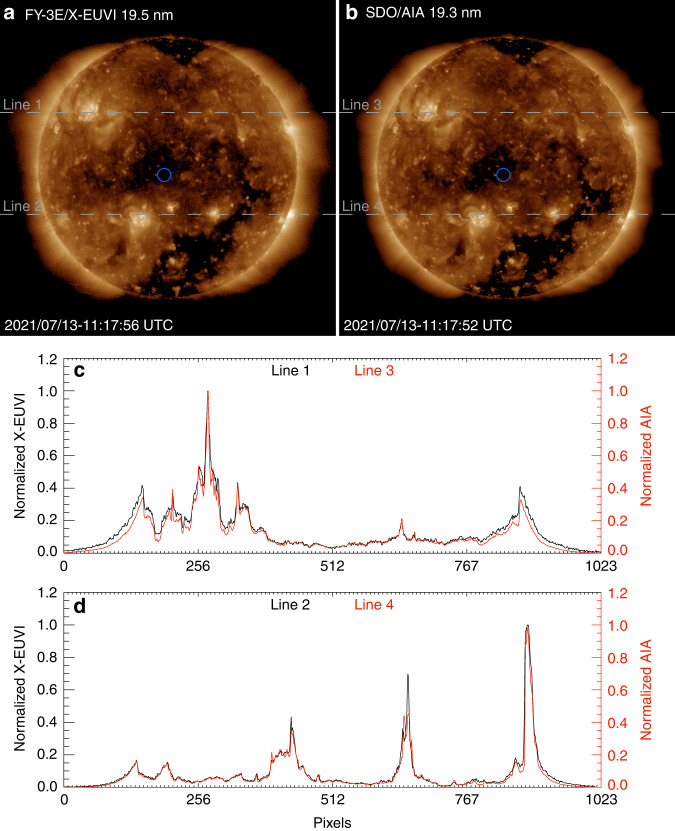
Comparison of EUV images from X-EUVI and AIA. **a** The positions of line 1 and line 2 from X-EUVI. **b** The positions of line 3 and line 4 from AIA. **c** The normalized grey value distributions of line 1 and line 3. **d** The normalized grey value distributions of line 2 and line 4

Due to the X-EUVI characteristic of a wide wavelength band from X-ray to EUV bands, the EUV images from X-EUVI should be comparable to the images from SDO/AIA, and the X-ray images should be comparable to those from Hinode/XRT at close times. The comparison of EUV images from X-EUVI and AIA is given in Fig. 5. The observation times of the two images are almost synchronous, and they are labelled in the lower left corner. Filaments, active regions and coronal holes can be clearly seen in both Fig. 5a (X-EUVI) and Fig. 5b (AIA). Moreover, Fig. 5c and d show the normalized grey value variations of the two types of images. Lines 1 and 2 are from X-EUVI, lines 3 and 4 are from AIA, and they are all marked in the image. Those morphologies are consistent with each other.

**Fig. 6:**
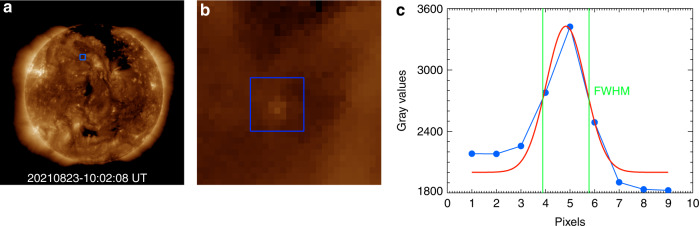
The observation of a point target to reveal the resolution of X-EUVI. The point target in **a** is marked in the blue rectangle, **b** is the enlarged view of the point target, and **c** is horizontal line across the peak value

The observation of a point target, shown in Fig. 6, is chosen to reveal the angular resolution of X-EUVI. The target is inside the blue rectangular boxes in both Fig. 6a and b, and Fig. 6b is a partially enlarged view of the blue box in Fig. 6a. The blue curve in Fig. 6c shows the grey values vs. pixel along the line which crosses the point target, and the red curve is the Gaussian fitting result. The green line marks the full widths at half maximum (FWHM) of the Gaussian distribution. The FWHM is <2 pixels, which means the angular resolution of X-EUVI EUV channel is better than the designed value 5.0″. Before launch, the angular resolution was measured by a EUV collimator at 19.5 nm. The tested result is two pixels equal to 5.0″ yet. For more detail, the measurement work will be described in the subsequent paper.

In addition, the blue circle areas in Fig. 5a and b, are chosen to evaluate the image signal-to-noise ratios of X-EUVI and AIA, respectively. The signal-to-noise ratios are respectively 5.8 times X-EUVI and 4.9 times AIA. They indicate that the ability of stray light suppression of X-EUV is similar to AIA.

**Fig. 7:**
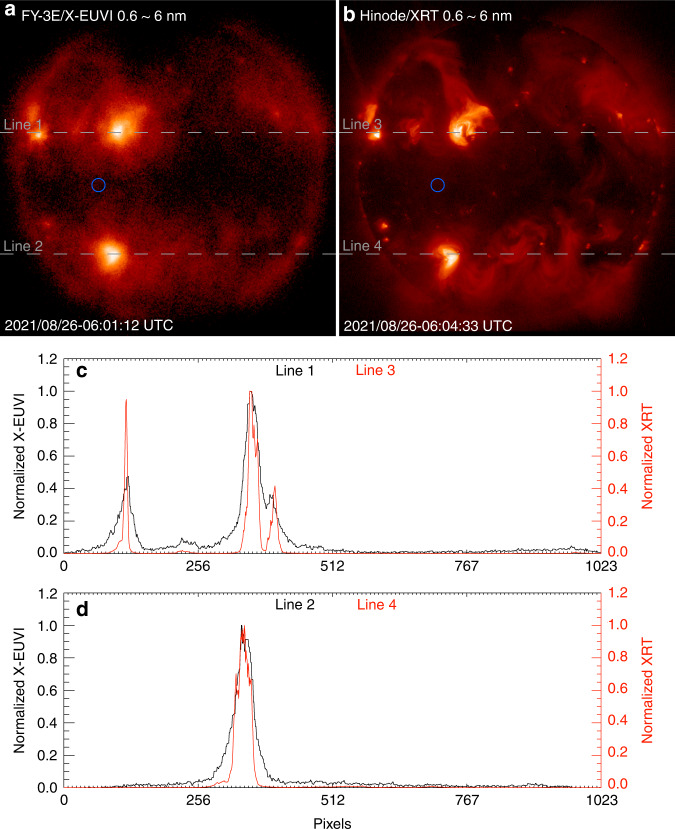
Comparison of X-ray images from X-EUVI and XRT. **a** The positions of line 1 and line 2 from X-EUVI. **b** The positions of line 3 and line 4 from XRT. **c** The normalized grey value distributions of line 1 and line 3. **d** The normalized grey value distributions of line 2 and line 4

**Fig. 8:**
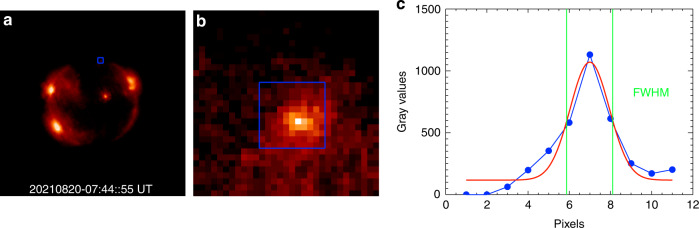
The observation of a point target to reveal the resolution of X-EUVI. The point target in **a** is marked in the blue rectangle, **b** is the enlarged view of the point target, and **c** is horizontal line across the peak value

Similarly, the comparison of X-ray images from X-EUVI and Hinode/XRT is shown in Fig. 7. Both morphological characteristics are consistent. Imaging a point target is also used to reveal the resolution of X-EUVI X-ray channels, which are shown in Fig. 8. Like Fig. 6, the FWHM is about 2.2 pixels, which means the angular resolution of X-EUVI X-ray channels is about 9.0″. We chose the weak fine structure to evaluate the angular resolution. Because there is some amount of scattering by the grazing incident mirror surfaces around the bright fine structure. It reduces the angular resolution of X-ray optics. In the next X-EUVI onboard the Fengyun-3J satellite, we are going to improve the roughness of the grazing incident mirror surfaces to decrease the scatter effect on the angular resolution. Before launch, the angular resolutions of X-EUVI X-ray channels were measured by a EUV collimator at 19.5 nm yet. The tested result is also two pixels equal to 8.2″. For more detail, it will be described in the subsequent paper too.

In addition, the blue circle regions in Fig. 7a and b, are chosen to evaluate the image signal-to-noise ratios. The signal-to-noise ratios are 2.7 times X-EUVI and 3.4 times XRT, respectively. They indicate that the ratios of X-EUV are similar to Hinode/XRT.

## Discussion

The development, calibration, and data processing algorithms for X-EUVI onboard the FY-3E satellite is described in this paper. This is the first space-based solar X-ray and extreme ultraviolet (EUV) imager for space weather and space physics in China. The preliminary results of in-orbit tests demonstrate that the performance of X-EUV satisfies the design requirements and the requirements of scientific investigations and operational forecasts for space weather. Before the scientific data release, the original solar data observed from X-EUVI will be processed by rotation correction, removal of noise, removal of the dark image, and flat field calibration. Then radiometric calibration will be carried out according to the solar irradiance data from the X-EUVS at the same time. After that, the solar images of X-EUVI will be processed further to generate continuous time-series produces of solar full-disk images. They can be displayed by the movie visualization such as 5 min, 1 h, 1 day, 27 days and so on. For space weather forecasts, they are not only suitable for observing short-time-scale changes in solar atmosphere such as flares but also suitable for monitoring long-lived features such as coronal holes. When the Sunbursts, they can be identified by finding the changes in the full disk and regional brightness of the time-series images. In addition, the threshold can be automatically calculated from the brightness distribution on a single image of 19.5 nm images and the coronal hole boundary can be determined and displayed automatically. Finally, the solar data with absolute solar brightness distribution will be released by the National Centre for Space Weather (NCSW)-China Meteorological Administration (CMA) according to its data policy at http://data.nsmc.org.cn/portalsite/default.aspx. Usually, the data will be released 2–3 h later. However, for special solar activity events, the period that the solar data released can be appropriately shortened.

Solar flares, coronal holes and filaments are typical structures on the solar surface in solar X-ray and EUV images that can be used to study the impact of solar activities on the thermosphere, ionosphere and magnetic field of the Earth, associated with X-ray and EUV irradiance measurements. The most important manifestation of solar activity is flaring. X-ray imaging observation data are particularly crucial for the study of high-temperature plasma in the corona (~10^7^ K), which represents the region with the strongest energy release and the core of solar flares. The EUV imaging can monitor low-coronal phenomena such as filaments in real-time and the CME phenomena. With the launch of X-EUVI on FY-3E, in association with auroral and ionosphere data from the Fengyun-3D Wide-field Auroral Imager (WAI) simultaneously, we will be able to systematically investigate the auroral and ionospheric dynamics and solar wind-magnetosphere–ionosphere couplings, including their hemispheric differences. This will also substantially improve our understanding of space weather and its accurate prediction.

Furthermore, a multispectrum EUV imager onboard the Fengyun-4C satellite and a dual-band WAI onboard the Fengyun-3H satellite have been developed in CIOMP for further space weather observations by CMA. The Fengyun-4C EUV imager has four bands of 9.4, 17.1, 21.1 and 30.4 nm and will be launched in 2025. The Fengyun-3H dual band WAI has two bands of 140–160 and 160–180 nm and will be launched in 2026. Combination observations of Fengyun-3D, FY-3E, Fengyun-3H and Fengyun-4C in both geosynchronous and sun-synchronous orbits will provide the space weather community with comprehensive data on solar activities and the near-Earth environment to study the Sun-Earth connection and its impact on the Earth space environment.

## Materials and methods

### Scientific objectives

The basic driver of solar activity exploration in China comes from the primary need of the NCSW of CMA with the single charge of operational specification and forecast of space environment conditions originating from the Sun and from the research needs of the space weather community with a focus on further study of the interaction of solar radiation storms, geomagnetic storms and radio blackouts associated with solar flares, coronal holes and solar X-ray and EUV radiation. Note that there are clear differences between the forecast-driven observational requirements of CMA and those of a purely research-oriented instrument. The instrument requirements of the X-EUV imager have to comply with these operational and scientific needs due to limited payload resources. After all, this X-EUVI will be the first space-based solar telescope to monitor solar flares and eruptions for the space weather community in China^27,28^.

Solar flares, coronal holes and filaments are typical structures on the solar surface in solar X-ray and EUV images that can be used to study the solar atmospheric structures, solar activity processes and their effect on the thermosphere, ionosphere and magnetic field of the Earth, associated with measurements of X-ray and EUV radiation. The most important manifestation of solar activity is flaring symbolizing the corona region with the strongest energy release, which is not visible from the ground due to the absorption of the Earth’s atmosphere. Observations of solar X-rays and EUV aid in the early detection of solar flares, CMEs, and other phenomena that impact the geospace environment. X-ray and EUV irradiance measurements provide a sensitive means of monitoring the beginning of solar flares—explosive events on the Sun’s surface associated with sunspot eruptions. X-ray imaging is used to monitor the hot outer atmosphere or corona of the Sun with high-temperature plasma in the corona (~10^7^ K). EUV imaging is used to observe and characterize complex active regions of the Sun, solar flares, and eruptions of solar filaments, which may give rise to CMEs, as well as the CME phenomena, originating from the part except for flare. EUV imaging of solar flares and solar eruptions can provide early warning of possible impacts on the Earth’s space environment and enable better forecasting of potentially disruptive events on the ground.

With the launch of the X-EUV imager on FY-3E to observe solar EUV and X-ray emissions for the next solar maximum, in association with auroral and ionosphere data from Fengyun-3D WAI simultaneously, both Fengyun-3D and FY-3E will operate in a polar-orbiting orbit and a sun-synchronous orbit, respectively, at an altitude of ~840 km with an ~99° inclination and an orbital period near 102 min. By the time of the launch of Fengyun-3H and Fengyun-4C near the next solar maximum, we will be able to comprehensively investigate the auroral and ionospheric dynamics and solar wind–magnetosphere–ionosphere couplings, including their hemispheric differences, systematically. This will also substantially improve our understanding of space weather and its accurate prediction.

The main scientific objectives for X-EUVI are as follows:

1. Image the solar disk and measure solar irradiance in the 19.5 nm EUV and 0.6–8.0 nm X-ray bands to observe the low-corona phenomenon and monitor and potentially forecast solar flares and eruptions.
2. Identify the active region size morphology and small-scale structures of the solar disk to investigate the physical changes of coronal holes and active regions.
3. Locate the positions of solar flares and coronal holes on the disk to forecast the magnitude of particle events and predict high-speed solar wind streams causing recurrent geomagnetic storms, respectively.
4. Monitor for changes indicating CMEs that may impact the Earth and cause geomagnetic storms. Large-scale, long-duration, possibly weakly emitting events and brightening of coronal filament arcades are used as evidence of CMEs.

According to these requirements, the X-EUVI instrument parameters are summarized in Table 2. The design will be analysed in detail in the following sections.

**Table 2:** Performance parameters of X-EUVI

Items	Parameters
Wavelength band	X-ray: X1 (0.6–8.0 nm); X2 (0.6–6.0 nm); X3 (0.6–5.0 nm); X4 (0.6–2.0 nm); X5 (0.6–1.6 nm); X6 (0.6–1.2 nm);
	EUV: 19.5 nm
Out-of-band response	X1: 0.7%; X2: 0.6%; X3: 0.7%; X4: 0.3%; X5: 1.4%; X6: 2.1%;
	EUV1: 4.1%; EUV2: 2.9%
Bandpass width	EUV: 0.7 nm
Exposure	10 ms–20 s
FOV	42.0′
Pixel resolution	X-ray: 4.1”; EUV: 2.5”
Temporal resolution	7–20 s
Pointing accuracy	3″
Stabilization	0.3″/20 s
Radiometric accuracy	15%

### Design of the solar X-ray and EUV imager

**Fig. 9:**
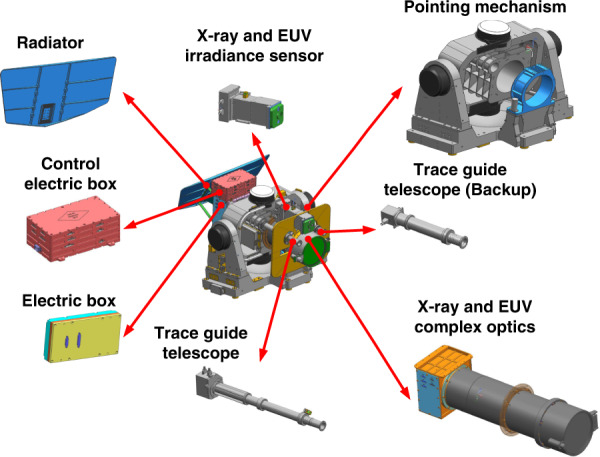
X-EUVI instrument and its composition

An innovative dual-band solar X-ray and EUV imager has been developed by CIOMP for observing the Sun, which is composed of X-ray and EUV complex optics, an X-ray and EUV irradiance sensor (X-EUVS), a compact Trace Guide Telescope (TGT), a pointing mechanism and an imaging stabilization system^29,30^. The instrument and its composition are shown in Fig. 9. The solar X-EUVI optics cover an X-ray band from 0.6 to 8.0 nm and a EUV band of 19.5 nm to observe solar X-ray and EUV images. X-EUVS monitors the solar absolute irradiance and calibrates the X-ray and EUV images. The TGT, pointing mechanism and imaging stabilization system constitute a servo system to capture and track the Sun and make X-EUVI point at the Sun accurately. The designs and analysis are described below.

### Design of dual-band optics in the X-ray and EUV regions

**Fig. 10:**
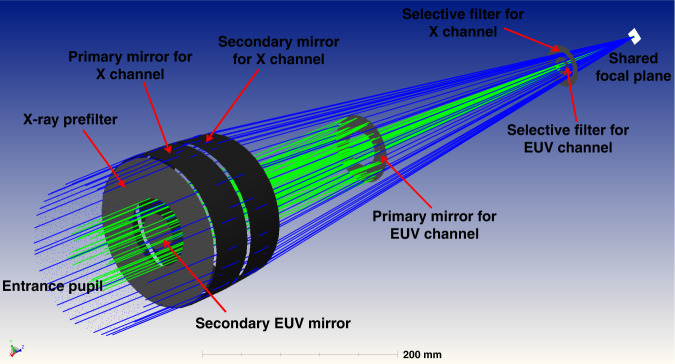
Optical path of dual-band optics in the X-ray and EUV regions

An innovative optics design is applied to X-EUVI, which employs complex X-ray and EUV optics in which EUV multilayer coating normal incidence optics occupy the centre of the X-ray grazing optics of the Wolter-I type. The two optics have a common axis and a common CCD detector, which are shown in Fig. 10. A band switching assembly is placed between the dual band optics and the common CCD detector, which is driven by a stepping motor. When the X-ray optic works, the X-ray port of the assembly is rotated to the corresponding position, and the border X-ray radiation passes through. When the EUV optics work, the EUV port of the switching assembly is rotated to its corresponding position, and the centre part radiation of the EUV band passes through. During X-EUVI operation in space, the X-ray port and EUV port are alternately switched to the X-ray optics and EUV optics, and dual-band images are obtained. The shortest period for the conversion between different ports is 0.3 s, and the shortest period for downloading the data of one frame image is 2 s. Therefore, X-EUVI can obtain solar X-ray and EUV images quickly for space weather forecasting and scientific investigations.

**Fig. 11:**
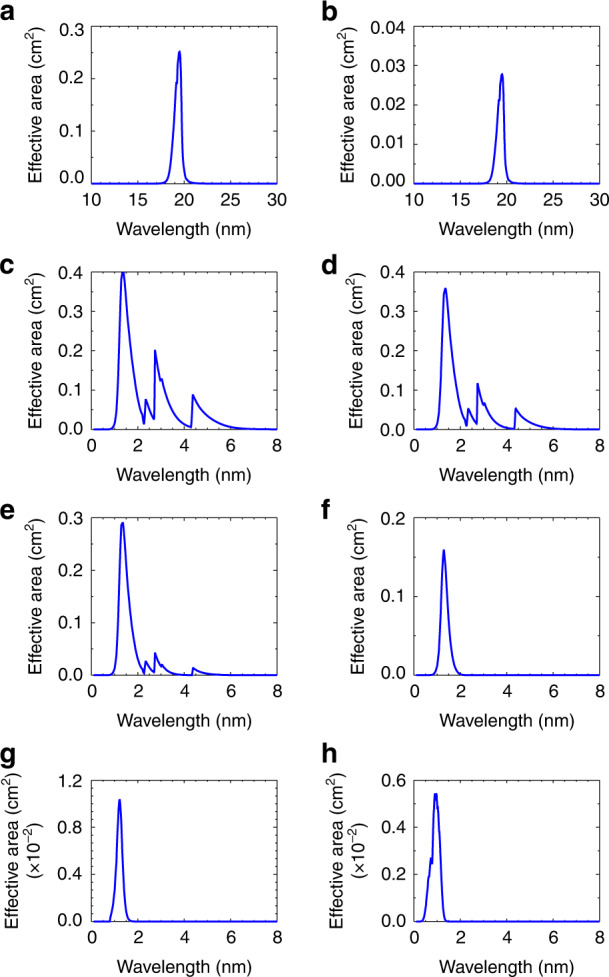
Effective area distribution of X-EUVI. **a** EUV1 band, **b** EUV2 band, **c** X1 band, **d** X2 band, **e** X3 band, **f** X4 band, **h** X5 band, and **g** X6 band

The X-ray grazing incidence optics employ Walter-I optics composed of a Zerodur superpolished mirror. With a grazing incidence angle of 1.74°, the Wolter-I optics have high reflectance from the X-ray to the visible wavelength band. To prevent visible and ultraviolet light over the 30 nm wavelength range from entering the optical system, a multilayer film filter of Al/Ti/Poly is applied to the pupil. The grazing incidence optics can also prevent higher-energy X-rays and other high-energy radiation from arriving at the CCD detector. A filter wheel assembly is placed in front of the CCD detector, which includes six pieces of X-ray filters and two pieces of EUV filters, to obtain the images in six X-ray bands and a EUV band. The effective area of the optical system represents the observation capacity of the X-ray optics, and their spectral distributions are shown in Fig. 11c–h. The effective area is equal to the product of the pupil area, the transmittance of the filters, the reflectivity of the mirrors and the quantum efficiency of the detector. The out-of-band rejection of each channel is calculated, and the values are 0.7% for the X1 band, 0.6% for the X2 band, 0.7% for the X3 band, 0.3% for X4 band, 1.4% for the X5 band, and 2.1% for the X6 band in the range of 0.1–35.0 nm.

The EUV optical system adopts a EUV multilayer coating normal incidence optical system, which is a two-mirror optic with a Mo/Si coating deposited on a superpolished substrate. The EUV multilayer coating mirrors have a high reflectivity of 44% and a narrow bandwidth of 0.7 nm. However, the EUV optics have higher reflectivity in the visible region, so a prefilter is installed in front of the optics to prevent the solar ultraviolet and visible light from entering the EUV optics. The prefilter consists of an aluminium film material, which has high transmittance in the region below 50 nm. There are two EUV filters on the filter assembly in front of the CCD detector, and the transmittance of the two differs by 12 times. When solar activity increases, the dynamic range of the EUV optical system can be increased by 12 times by switching the EUV filter. The effective areas of the EUV optics are also shown in Fig. 11a and b. The out-of-band rejections of the EUV optics are 4.1% for the EUV1 band and 2.9% for the EUV2 band in the range of 0.1–35.0 nm.

### Design of the solar X-ray and EUV irradiance sensor

X-EUVI is equipped with X-EUVS, consisting of an X-ray irradiance sensor and a EUV irradiance sensor, for observing solar irradiance and calibrating X-ray and EUV images. X-EUVS has almost the same response distributions as X-EUVI and is sensitive enough to measure solar irradiance in orbit and calibrate the radiation response of X-EUVI. More details of the design are as follows.

**Fig. 12:**
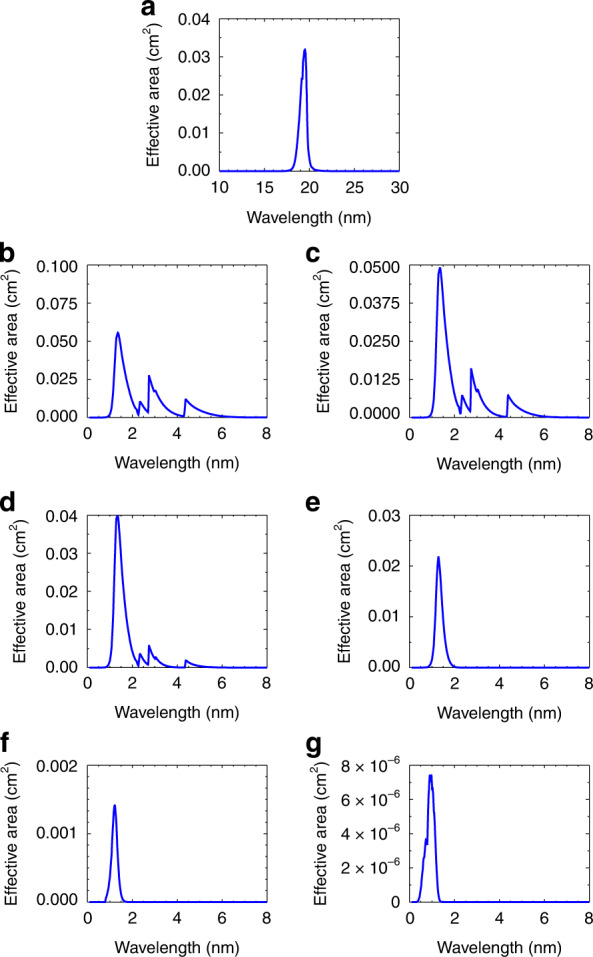
Effective area distributions of X-EUVS. **a** EUV band, **b** X1 band, **c** X2 band, **d** X3 band, **e** X4 band, **f** X5 band, and **g** X6 band

The innovative type of solar X-ray irradiance sensor is designed for solar X-ray irradiance measurement and consists of an entrance filter, an X-ray grazing component, a filter wheel assembly, and an X-ray silicon diode. The entrance filter is located in front of the X-ray irradiance sensor to prevent sunlight over 30 nm from entering the sensor. The grazing component focuses the solar X-rays on the silicon diode and prevents the high-energy radiation from going into the diode. The bandpass filters on the filter wheel assembly in front of the diode allow only the special bands of X-rays to enter the sensor. The field of view (FOV) of the irradiance sensor is large enough to cover the full-disk Sun. The X-ray band spectral responses of X-EUVS are almost the same as those of X-EUVI. The spectral distributions of the X-ray irradiance sensor effective area, which is equal to the product of the pupil area, the transmittance of the filters, the reflectivity of the mirrors and the quantum efficiency of the diode, are shown in Fig. 12b–g.

The EUV irradiance sensor is developed for solar EUV irradiance measurement and consists of a prefilter, EUV multilayer coating mirrors and a EUV silicon diode. The pre-filter can filter out sunlight exceeding 50 nm. Only 19.5 nm EUV light can be focused on the silicon diode after the EUV narrow passband multilayer mirrors. Then, the diode converts EUV photons of 19.5 nm to electrons. The EUV irradiance sensor employs the same prefilter and the same EUV multilayer coatings as the X-EUVI EUV imaging optics. Thus, the spectral response is the same as that of the EUV imaging optics. The spectral distribution of the EUV irradiance sensor effective area is also shown in Fig. 12a.

### High-precision pointing, tracking and imaging stabilization system

X-EUVI is installed on the Sun-synchronous orbit (twilight) satellite FY-3E, which has an orbital altitude of 836 km and an orbital period of 102 min. Since FY-3E is a three-axis stabilized spacecraft with respect to the Earth, the position of the Sun changes in the X-EUVI coordinate system in real-time. Therefore, X-EUVI should have the function of tracking the Sun. In addition, to eliminate residual jitter after spacecraft attitude control and instrument Sun tracking, X-EUVI should have an image stabilization function.

To ensure that X-EUVI can capture, track and image the Sun stably, a two-degree-of-freedom tracking and image stabilization system has been developed. When X-EUVI works in orbit, the two-degree-of-freedom tracking mechanism first roughly points to the Sun according to the solar position data of the spacecraft. Then, X-EUVI precisely points to the Sun using a lock-in control system based on TGT solar position data. The pointing accuracy is 3”.

**Fig. 13:**
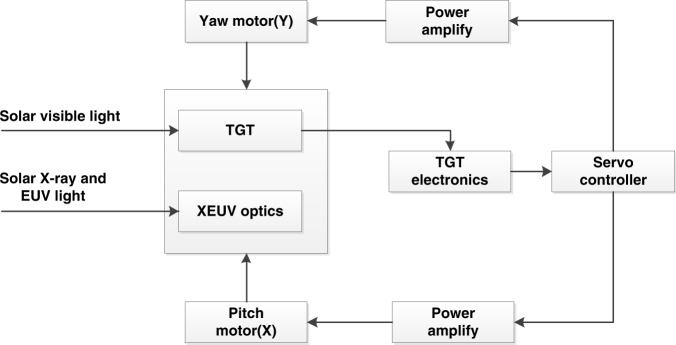
Schematic diagram of the Sun tracking mechanism

**Fig. 14:**
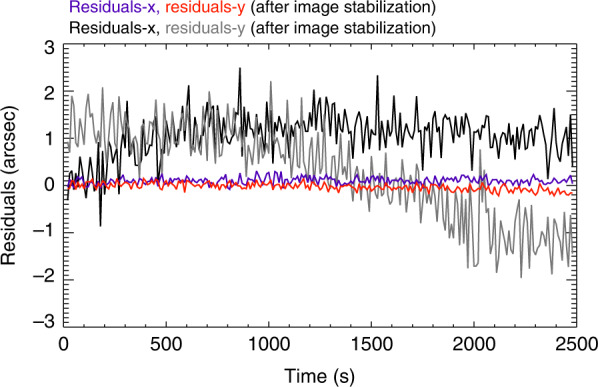
Residuals before and after image stabilization

In addition to the pointing deviation, the high-frequency jitter from the satellite platform affects the X-EUVI imaging performance. Therefore, an image stabilization table is employed to tilt the secondary mirror of the EUV optics to compensate for the image jitter caused by the high-frequency vibration. The image stabilization mechanism is an open-loop control system. After tracking the Sun, the image stabilization software samples the TGT signal every 1 ms and uses this signal to calculate the angle compensation of the secondary mirror. Four piezoelectric ceramics (PZT) tilt the secondary mirror to the calculated angle according to the position sensor integrated into the secondary mirror. Each PZT can tilt the secondary mirror by ±24 arcsecs in the focal plane of the EUV optics. A schematic diagram of the Sun tracking mechanism is shown in Fig. 13 and Fig. 14 shows the time profiles of the calculated residual error measured during the initial performance test after launch. The on-orbit performance of the image stabilization mechanism shows that the pointing stability is 0.3″.

### Calibration

X-EUVI will work in orbit over 6 years, and the instrument performance will be degraded by strong and long-term solar X-ray and EUV radiation. To obtain solar X-ray and EUV absolute brightness images, X-EUVI requires periodic full-field radiometric calibration.

### Flat-field calibration

Since the FY-3E satellite rotates at a constant speed relative to the Sun, the solar images taken by X-EUVI are also uniformly rotated relative to the centre of the FOV. The instrument can be calibrated by multiple rotation solar images. Space-based solar observation instruments usually utilize the KLL algorithm to carry out flat-field calibration in orbit. The KLL algorithm does not rely on experimental equipment, directly using the offset images to calculate the flat-field matrix of the imagers, which is widely used in SDO/AIA, SOHO/EIT and other instruments. This paper proposes an improved KLL algorithm based on rotation images to compute the X-EUVI flat-field matrix. The algorithm includes the determination of the solar disk centre, calculation of the rotation, image coordinate transformation, the KLL algorithm and image restoration. Since the temporal resolution of X-EUVI is <10 s, the morphological features of two adjacent solar images can reasonably be assumed to remain unchanged during the calculation; thus, only translation and rotation exist. Although the instrument moves around the Earth at a uniform speed on board the FY-3E satellite, the geometric centre between two consecutive images may shift due to the instantaneous vibration of the satellite, and the rotation angle is not equal to the rotation angle of the satellite around the Earth. Therefore, the geometric centre is directly calculated by the Sobel operator, and the rotation angle is calculated by the log-polar transform and related algorithm. Since solar images are continually rotating, the imager flat-field matrix can be acquired at any time when the instrument is working.

### Radiometric calibration

Through measurement of the Sun’s full-disk irradiance and flat-field calibration based on rotating solar images, X-EUVI can perform on-orbit radiometric calibration. Before launching, X-EUVS is calibrated in CIOMP. First, the parameters of the sensor optics are calibrated by an X-ray and EUV reflectometer, which include the transmittance of the prefilter, transmittance of the passband filter and reflectivity of the mirror. The pupil area of the sensor is measured. The diode response coefficient is calibrated by an AXUV100G transfer standard detector, which has been calibrated by PTB. Then, using these parameters, the irradiance-current conversion factor for each channel of X-EUVS is calculated, and the irradiance of the full-disk Sun can be calculated by the formula (1).

1 $$E_{{\rm {sun}}} = \frac{{I_{\rm {d}}}}{{R_d \cdot A \cdot \tau }}({\rm {W}} \cdot {\rm {m}}^{ - 2})$$

where *E*_sun_ is the solar full-disk irradiance, *I*_d_ is the photon current tested by the sensor, *R*_*d*_ is the response factor of the diode, *A* is the entrance pupil area, and *τ* is the system transmittance, which is the product of the filter transmittance and mirror reflectivity. According to the parameters of X-EUVS, the instrument irradiance-current conversion factor $\frac{{I_{\rm {d}}}}{{R_d \cdot A \cdot \tau }}$ for each band is calculated, as shown in Table 3.

**Table 3:** Current-irradiance conversion factors of the X-ray and EUV sensor

Channel	Current–irradiance conversion factor (W·m^−2^ • pA^−1^)
X1 of 0.6–8.0 nm	2.97E−06
X2 of 0.6–6.0 nm	2.71E−06
X3 of 0.6–5.0 nm	2.27E−06
X4 of 0.6–2.0 nm	2.27E−06
X5 of 0.6–1.6 nm	1.54E−06
X6 of 0.6–1.2 nm	8.84E−06
EUV of 19.5 nm	4.75E−06

During on-orbit radiometric calibration, the total solar disk irradiance in the EUV and X-ray regions will be measured by X-EUVS. Meanwhile, solar X-ray and EUV images will be taken by X-EUVI and corrected by the flat-field matrix. All pixel values of a flat-field corrected solar image will be accumulated and divided by the solar irradiance measured by X-EUVS, and the unit pixel value irradiance will be obtained. The solar irradiance of a pixel can be obtained by multiplying the pixel value by the unit pixel value irradiance. The absolute brightness of the pixel can be obtained by dividing the pixel irradiance by its solid angle, as calculated in formula (2). Then, solar X-ray and EUV images with absolute irradiance are obtained.

2 $$B_{\rm{pix}} = \frac{{E_{\rm{sun}}}}{{{\rm{DN}_{\rm{sun}}}}} \cdot \frac{{{{\rm{DN}}_{{\rm{pix}}}}}}{\omega }$$

where *E*_sun_ is the disk solar irradiance obtained by X-EUVS, DN_sun_ is the pixel value sum of a solar image observed by X-EUVI, DN_pix_ is the pixel value of the pixel, *ω* is the solid angle of the pixel, and *B*_pix_ is the calibrated brightness of the pixel.
